# Clinical characteristics and outcome in patients with anti-AMPAR2 encephalitis: a case series and literature review

**DOI:** 10.3389/fnhum.2025.1586005

**Published:** 2025-07-15

**Authors:** Congcong Wang, Fanyu Kong, Xianwen Yu, Yan Li, Meihe Yang, Zhihua Si, Rutao Liu, Ke Hu, Bing Yang

**Affiliations:** ^1^Internal Medicine of Traditional Chinese Medicine, Shandong University of Traditional Chinese Medicine, Jinan, Shandong, China; ^2^Department of Neurology, Shandong Provincial Qianfoshan Hospital, The First Affiliated Hospital of Shandong First Medical University, Jinan, Shandong, China; ^3^Shandong Institute of Neuroimmunology, Jinan, Shandong, China; ^4^Shandong Provincial Medicine and Health Key Laboratory of Neuroimmunology, Jinan, Shandong, China; ^5^Department of Neurology, Zibo Central Hospital, Zibo, Shandong, China; ^6^Department of Emergency Medicine, Shandong Provincial Qianfoshan Hospital, The First Affiliated Hospital of Shandong First Medical University, Jinan, Shandong, China

**Keywords:** anti-alpha-amino-3-hydroxy-5-methyl-4-isoxazolepropionic acid receptor encephalitis, limbic encephalitis, simple amnesia, autoimmune encephalitis, immunotherapy, outcome

## Abstract

Anti-alpha-amino-3-hydroxy-5-methyl-4-isoxazolepropionic acid receptor encephalitis is a rare autoimmune encephalitis, with only a few series describing its typical clinical manifestations and prognosis. Here, we present three newly identified patients with anti-AMPAR encephalitis and were followed up for prognostic evaluation. The mean age of the patients was 47 years (range, 32–57). All three patients experienced memory issues, with two showing signs of typical limbic encephalitis (LE). Cranial magnetic resonance imaging (MRI) in two patients demonstrated lesions in the bilateral temporal lobes, insula, and cingulate gyrus as well as significant cortical atrophy after 1 month of follow-up. No acute lesions were observed on cranial MRI in the third patient at the onset of symptoms. One patient had positive antibodies for both AMPAR1 and AMPAR2 in cerebrospinal fluid (CSF), while the other two patients only had positive antibodies for AMPAR2. Severe clinical symptoms and high CSF antibody levels were found in two patients. Immunotherapy demonstrated partial efficacy in all three patients. Two patients exhibited favorable responses to first-line immunotherapy. In contrast, the third patient experienced a suboptimal response to the initial treatment, with no remission and subsequent disease relapse. Following second-line immunotherapy, her condition stabilized; however, she continued to suffer from significant cognitive impairment. One patient was diagnosed with a viral infection, but no tumors were found in any patients. Besides its typical manifestation as LE, anti-AMPAR2 encephalitis may also present as simple amnesia. It is advised to monitor CSF antibodies and their level changes. While first-line immunotherapy is partially effective, some patients may need additional second-line therapy. Viral infections could be a predisposing factor; thus, routine CSF virus testing is recommended. Additionally, screening for tumors and follow-up assessments are also important.

## 1 Introduction

Autoimmune encephalitis is a broad term representing a spectrum of encephalitis conditions mediated by autoimmune mechanisms, with paraneoplastic AE referring to cases of AE combined with tumors (Graus et al., 2004). After the initial identification of anti-N-methyl-D-aspartate receptor (NMDAR) encephalitis in 2007 (Dalmau et al., 2007), numerous autoantibodies against neuronal cell surface or synaptic proteins have been reported. Among the various AE, anti-NMDAR encephalitis is the most prevalent (approximately 54%–80% of AE cases) (Ren et al., 2021), followed by anti-leucine-rich glioma inactivated protein 1 (LGI1) encephalitis and anti-gamma-aminobutyric acid type B receptor (GABABR) encephalitis (Davies et al., 2010; Guan et al., 2016; Suh-Lailam et al., 2013). In 2009, anti-alpha-amino-3-hydroxy-5-methyl-4-isoxazolepropionic acid receptor (AMPAR) encephalitis was first reported, which is an AE associated with antibodies targeting the GluA subunit of the AMPAR (Lai et al., 2009). AMPAR, a glutamate-gated ion channel, consists of the tetramer GluA1-4 and is widely expressed in the central nervous system (CNS), notably in the hippocampus and limbic regions. Limbic encephalitis (LE) is the most common symptom of anti-AMPAR encephalitis, characterized by short-term memory loss, confusion, and memory deterioration (Zhang T. Y. et al., 2021). Some individuals could experience hallucinations, movement problems, and additional neurological symptoms. In recent years, there has been a rapid increase in case reports of anti-AMPAR encephalitis. However, the disease remains rare, with just over 100 cases reported in the literature to date (Zhu et al., 2024). An underlying tumor is present in 80.9% of patients, with thymoma being the most frequent, followed by lung cancer (Zhu et al., 2024). Even though the disease responds well to immunotherapy, the long-term prognosis is influenced by paraneoplastic antibodies and associated symptoms or tumors (Höftberger et al., 2015). Here, we report on three cases of anti-AMPAR2 encephalitis, showcasing typical clinical symptoms, imaging features, and follow-up imaging and prognostic data, which offer important insights for effective diagnosis and treatment of the disease.

## 2 Materials and methods

**FIGURE 1:**
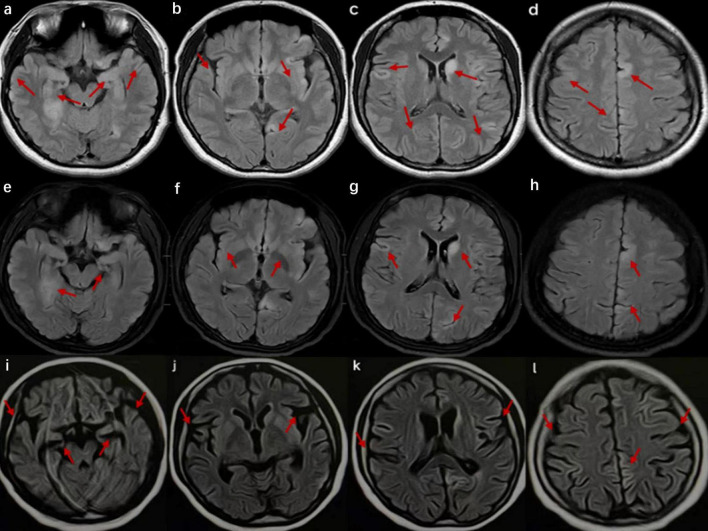
Cranial magnetic resonance imaging (MRI) of the patient in case 1. **(a–d)** Images at the time of admission show high signals in the bilateral temporal lobes, hippocampus, insula, frontal lobes, parietal lobes, and cingulate gyrus and on T2 FLAIR of the left caudate nucleus. **(e–h)** Images after 2 weeks of hospitalization exhibit a slight decrease in the extent of the abnormal signals compared to previous images. **(i–l)** Images taken 2 months after onset reveal multiple abnormal signals, indicating brain atrophy.

**FIGURE 2:**
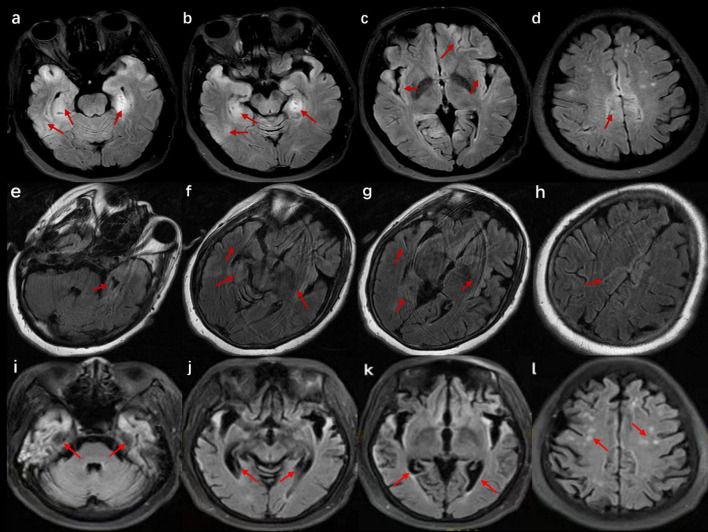
Cranial magnetic resonance imaging (MRI) of the patient in case 3. **(a–d)** Images at the time of admission show bilateral T2 fluid-attenuated inversion recovery (FLAIR) high signals in the hippocampus, insula, cingulate gyrus, and cerebral cortex. **(e–h)** Images after 2 weeks of hospitalization suggest a reduced extent of abnormal signals compared with previous scans. **(i–l)** Images after 4 months of onset reveal severe brain atrophy with multiple high signals on T2 FLAIR.

In this study, three patients diagnosed with anti-AMPAR2 encephalitis from March 2022 to June 2023 were included, and they all met the autoimmune encephalitis diagnostic criteria published in 2016 (Graus et al., 2016). All three patients had AMPAR2 antibodies in their cerebrospinal fluid (CSF) detected through a cell-based assay (CBA; Jiangsu Simcere Diagnostic Laboratory). Collected data included the clinical features, laboratory test findings, neuroimaging examination results, treatment, and prognosis of the patients. The image results are detailed in Figures 1, 2. The clinical features, treatment, and outcome of patients are listed in Table 1. The case reports, reviews, and cohort studies of anti-AMPAR encephalitis were reviewed.

**TABLE 1:** Clinical features, treatment, and outcome of patients with anti-alpha-amino-3-hydroxy-5-methyl-4-isoxazolepropionic acid receptor (AMPAR) encephalitis.

Patient	1	2	3
Sex (M/F)	F	M	F
Age (years)	32	57	52
**Symptoms at presentation**			
Memory impairment	+	+	+
Cognitive decline	+	–	+
**CSF**			
WCC (mm^3^)	2	5	6
Protein (g/L)	0.3	0.28	0.22
Pathogen	HSV	NA	NA
**Anti-AMPAR1 antibodies**			
Serum	–	NA	–
CSF	–	1:10	–
**Anti-AMPAR2 antibodies**			
Serum	1:1000	NA	1:100
CSF	1:100	1:10	1:100
**Cranial MRI**			
Upon admission	Abnormal (cortical and basal ganglia lesions)	Normal	Abnormal (cortical lesions)
Follow-up after discharge	Abnormal (brain atrophy)	Normal	Abnormal (brain atrophy and centrum semiovale lesions)
Tumor	–	–	–
**Therapy**			
First-line immunotherapy	Steroids; IVIG	Steroids	Steroids; IVIG
Second-line immunotherapy	–	–	Rituximab
Outcome	Full recovery	Full recovery	Severe cognitive impairment

AMPAR, alpha-amino-3-hydroxy-5-methyl-4-isoxazolepropionic acid receptor; CSF, cerebrospinal fluid; IVIG, intravenous immunoglobulin; MRI, magnetic resonance imaging; NA, not applicable; WCC, white cell count.

Informed consent was obtained from the families of the patients for the inclusion of their data in the study. Approval for the study was granted by the Ethics Committee at the First Affiliated Hospital of Shandong First Medical University.

## 3 Case presentation

### 3.1 Case 1

A 32-year-old previously healthy woman was admitted to the hospital because of fever and sleep disturbances, accompanied by memory loss. Ten days before hospitalization, the patient developed a fever, nasal congestion, and a runny nose, along with obvious difficulty in sleeping, generalized weakness, and nighttime irritability. Furthermore, the patient exhibited memory loss of recent events and verbal expression problems 1 week prior to hospitalization. The neurological examination showed fuzzy consciousness, impaired verbal expression, memory deficit for recent events, and slow reaction time, but essentially normal comprehension and computation abilities, as well as normal muscle strength and tone in the extremities. During hospitalization, the fuzzy consciousness and speech disorder of the patient progressively worsened, with the development of involuntary movement of the right limbs. Ultimately, the patient lost the ability to walk and speak. The patient was incapable of cooperating with the Mini-Mental State Examination (MMSE) and the Montreal Cognitive Assessment (MoCA) scale examinations. Lumbar puncture revealed an initial pressure of 240 mmH_2_O, with colorless and clear CSF, and normal levels of white cell count (WCC), protein, glucose, and chloride (Table 1). Albumin quotient (QALB) was 4.11. Serum and CSF tests for AE-related antibodies indicated positive anti-AMPAR2 antibodies, with serum at 1:1000 + and CSF at 1:100 + (Table 1). Additionally, next-generation sequencing of CSF detected the presence of herpes simplex virus (HSV). Magnetic resonance imaging (MRI) of the brain indicated patchy abnormal signals in the left basal ganglia region and swelling in some cerebral gyri of the bilateral cerebral hemispheres, left caudate nucleus head, and bilateral hippocampus (Figure 1). No malignancies were found in the computed tomography (CT) scans of the chest, abdomen, and pelvis, and the comprehensive positron emission tomography-computed tomography (PET/CT) scans of the entire body showed no significant metabolic increases.

The patient received an intravenous injection of methylprednisolone (IVMP) with the following dosage: 500 mg daily for 3 days, followed by 250 mg daily for another 3 days, and finally reduced to 80 mg daily for 2 weeks. In addition, the patient received intravenous acyclovir (0.5 g every 8 h for 10 days) and intravenous immunoglobulin (IVIG, 0.4 g/kg/day for 5 consecutive days). Two weeks later, the patient’s cognitive function showed no significant improvement, and her condition continued to deteriorate. However, repeated brain MRI suggested a slightly better extent of the lesions in the left caudate nucleus head, some cerebral gyri in the bilateral cerebral hemispheres, and bilateral hippocampal regions compared to the previous MRI findings (Figure 1).

Two months post-onset, the outpatient follow-up revealed that the patient was still unable to speak and experienced no significant improvement in cognitive function, scoring 12 on the MMSE and 10 on the MoCA assessments. The brain MRI indicated that multiple abnormal signals had improved, but brain atrophy was detected (Figure 1). Once discharged from the hospital, steroids decrease over time. One year after discharge, the patient stopped using prednisone. At the 1.5-year follow-up, she demonstrated recovery without evident clinical symptoms, with an MMSE score of 27 and a MoCA score of 26. However, tumor screening was not performed, despite being recommended.

### 3.2 Case 2

A 57-year-old male patient with a history of diabetes mellitus presented with memory loss prior to hospitalization. Four days before admission, the patient experienced memory impairment, characterized by an inability to recall recent events even with prompts. However, no other mental or behavioral abnormalities were reported. Neurological examination indicated only memory loss. The patient’s MMSE score was 27, while his MoCA score was 24. Lumbar puncture results indicated an initial CSF pressure of 150 mmH_2_O, with normal WCC, protein, glucose, and chloride levels (Table 1). Screening for AE-associated antibodies revealed the presence of CSF anti-AMPAR1 antibody at a dilution of 1:10 and anti-AMPAR2 antibody at a dilution of 1:10 (Table 1). Brain MRI examination showed no abnormalities. The EEG shows slow wave rhythms in a multifocal distribution. Consequently, the patient underwent treatment with methylprednisolone at a dosage of 120 mg per day for 5 days, and the dosage was gradually reduced. He also received citicoline to improve memory loss. After 2 weeks of therapy, the patient showed no worsening of memory loss and exhibited no other clinical manifestations. Outpatient follow-up 1 month later revealed that he was in a stable condition, scoring 28 on the MMSE and 25 on the MoCA. Brain MRI also indicated no significant changes. After a year, the patient discontinued the prednisone. He exhibited a successful recovery, with no indications of memory impairment, reflected by an MMSE score of 30 and a MoCA score of 29, and tumor screening confirmed the absence of tumors.

### 3.3 Case 3

A 52-year-old woman was admitted to the hospital because of memory loss. A week before being admitted, the patient exhibited memory loss, unresponsiveness, decreased speech ability, a dull expression, and limb weakness, all without any obvious cause. Two days before admission to our hospital, these symptoms worsened and were accompanied by a fever of 38°C. Further physical neurological examination revealed diminished speech function, unresponsiveness, and apathy, while limb muscle strength and tone were normal. The lumbar puncture test demonstrated an initial pressure of 170 mmH_2_O, along with normal WCC and normal levels of protein, glucose, and chloride (Table 1). Multiple abnormal signals were detected in the hippocampus, insula, cingulate gyrus, and cerebral cortex on the brain MRI (Figure 2). The possibility of a viral infection remains. Acyclovir 0.5 g was given intravenously every 8 h until the autoimmune encephalitis antibody test results were obtained. The patient was positive for anti-AMPAR2 antibodies in both serum and CSF, with a dilution of 1:100 (Table 1). The patient received IVMP (methylprednisolone 500 mg daily for 3 days, followed by 250 mg daily for another 3 days, and then 80 mg daily for 2 weeks) and IVIG (0.4 g/kg daily for 5 days). Further refinement of the chest, abdominal, and pelvic CT scans did not reveal any tumor-occupying manifestations. After 22 days of hospitalization, the patient exhibited no apparent improvement in cognitive function, as indicated by an MMSE score of 12 and a MoCA score of 9. Repeated brain MRI demonstrated multiple abnormal signals with reduced extent of the lesions (Figure 2).

Four months after symptoms began, during an outpatient follow-up, the patient still exhibited notable cognitive and language impairments, with an MMSE score of 13 and a MoCA score of 10. Brain MRI showed brain atrophy and multiple abnormal high signals. After a lengthy rehabilitation process, she regained some language function but continued to have cognitive difficulties. Due to a recurrence 1 year ago, the treatment was switched to rituximab 500 mg every 3 months at another hospital. She still experiences severe dementia and cannot take care of herself. In addition, no tumors were detected during the yearly tumor screening.

## 4 Discussion

Anti-AMPAR encephalitis is a recently discovered AE characterized by an acute or subacute onset of neurological symptoms. An upward trend in the incidence of AE has been observed due to improved detection and enhanced clinical knowledge. A retrospective study in Israel found that the rate of AE cases among yearly hospital admissions increased significantly, from 3.8 per 100,000 in 2010 to 18.8 per 100,000 in 2020. AE makes up 35% of patients with encephalitis (Segal et al., 2024). However, anti-AMPAR encephalitis is rare, accounting for 1%–2% of AE cases.

Excitatory neurotransmission in the brain is primarily mediated by glutamate, and its receptors are usually divided into ionotropic and metabotropic types. Moreover, ionotropic glutamate receptors (iGluRs) are divided into three main receptor families: AMPAR, NMDAR, and kainate receptors (KAR). Although all three types of receptors bind to a common endogenous ligand, they exhibit certain differences in their pharmacology, biochemistry, and regulation (Mayer, 2005). Among them, the AMPARs are iGluRs with high affinity and consist of homo- or hetero-oligomers of four subunits, namely GluA1-4 (Boulter et al., 1990; Keinänen et al., 1990). AMPARs are found at relatively higher levels in the hippocampus and other limbic regions.

Anti-alpha-amino-3-hydroxy-5-methyl-4-isoxazolepropionic acid receptor 1 and AMPAR2 antibodies can specifically bind to the extracellular domains of the subunits GluA1 and GluA2, forming antibody-receptor complexes. This action prompts receptor aggregation and cross-linking, allowing entry into the cell via a reticin-dependent endocytic pathway, which results in the internalization of AMPAR and its delivery to lysosomes for degradation (Peng et al., 2015). Given that AMPARs are essential for synaptic transmission in neurons, a decline in their quantity can diminish synaptic effectiveness and negatively affect hippocampus-dependent short-term memory. Furthermore, changes in the ratio of GluA1/GluA2 on the neuronal surface can influence neural activity. When the GluA1 subunit is excessively activated, it can lead to an increase in calcium ion influx in neurons, resulting in cellular excitotoxicity. By binding directly to the AMPAR subunit and regulating its expression, the Sorbs2 protein can elevate the GluA1/GluA2 ratio, increase neuronal hyperexcitability, and consequently trigger epileptic seizures (Ban et al., 2024). This mechanism may play an important role in the pathophysiological process of anti-AMPAR encephalitis, offering a potential target for the treatment of this disease.

Patients with anti-AMPAR encephalitis often exhibit a combination of tumors. Zhu et al. analyzed 94 patients, of whom 80.9% had tumors. These tumors included thymoma (43.4%), lung cancer (30.3%), breast cancer (11.8%), and ovarian tumors (7.9%) (Zhu et al., 2024). Additionally, medullary thyroid cancer, bladder cancer, melanoma, and Ewing’s sarcoma have been reported in a few patients (Samad and Wong, 2018; Zhang T. Y. et al., 2021).

Anti-AMPAR encephalitis is significantly correlated with thymoma, particularly in patients with invasive thymoma. The invasive or metastatic nature of thymoma might further promote the autoimmune response, but there is no direct connection between the degree of malignancy of thymoma and the specific type of anti-AMPAR encephalitis (Guasp et al., 2021). The presence of thymoma can lead to a deficiency in central immune tolerance, which may activate autoreactive T and B cells, resulting in an autoimmune response against neuronal surface antigens (Guasp et al., 2021). Lung cancer is frequently linked to paraneoplastic neurologic syndromes (PNSs), such as anti-Hu antibody encephalitis, and the presence of anti-AMPAR antibodies might indicate the presence of tumors (Zhang T. Y. et al., 2021). Through molecular mimicry mechanism, tumor antigens may trigger an autoimmune response against AMPAR, potentially leading to damage in the CNS. The tumor microenvironment may promote inflammatory cell infiltration and antibody production, with anti-AMPAR antibodies potentially aggravating neuronal dysfunction (Ricken et al., 2021).

Some anti-AMPAR encephalitis patients have a history of viral infection (Zhang T. Y. et al., 2021). The relationship between viral infections in the CNS and related autoimmune reactions is complex. Through the detection of herpes virus polymerase chain reaction (PCR) positive CSF samples, Linnoila et al. identified a patient who simultaneously had HSV-1 DNA in the initial sample, while anti-AMPAR antibodies were detected later. In another case, a patient initially diagnosed with HSV-1 encephalitis later showed positive anti-AMPAR antibodies (Linnoila et al., 2016). These findings suggest that HSV-1 infection can not only cause typical viral encephalitis but also induce autoimmune responses against non-NMDAR targets, such as AMPAR, through mechanisms such as molecular mimicry, neuronal antigen exposure due to inflammation-mediated disruption of the blood-brain barrier (BBB), or an imbalance of immune homeostasis following infection (Linnoila et al., 2016).

After HSV infection, the innate immune system initiates a multi-dimensional response mechanism through the Toll-like receptor (TLR) family. Through the recognition of the viral envelope glycoproteins B (gB), gC, and gD, TLR2 activates downstream signaling pathways and induces the release of pro-inflammatory factors interleukin-6 (IL-6) and tumor necrosis factor-α (TNF-α) (Jahanban-Esfahlan et al., 2019). TLR9 plays an important role in exacerbating neuronal damage by binding to the unmethylated cytosine-phosphate-guanine (CpG) DNA of the virus (Jahanban-Esfahlan et al., 2019). By recognizing the double-stranded RNA (dsRNA) produced during HSV replication, TLR3 can significantly hinder viral replication by inducing the expression of type I interferon (Jahanban-Esfahlan et al., 2019). Persistent viral invasion and excessive inflammatory response can disrupt the integrity of the BBB, promoting the infiltration of peripheral inflammatory cells and the spread of the virus to the brain tissue. The adenosine triphosphate (ATP) released by infected neurons activates the purinergic receptor P2Y12 on the surface of microglia, driving their directional migration to the site of infection and further promoting the secretion of inflammatory mediators such as C-C motif chemokine ligand 2 (CCL2), CCL5, IL-6, and TNF-α, thereby forming a cascade amplification effect (Liao et al., 2025). Antigen exposure resulting from nerve tissue damage may activate autoreactive B cells, leading to the production of autoantibodies against the surface antigens of neurons (Gnann and Whitley, 2017).

Herpes simplex virus infection may induce the production of anti-AMPAR antibodies through molecular mimicry mechanism. Specifically, certain HSV proteins share structural similarities with AMPAR regions in the brain, leading the immune system to mistakenly target AMPAR during the antiviral response. Additionally, the neuroinflammatory response triggered by HSV may further enhance AMPAR antibody production and contribute to neuronal damage, affecting patient outcomes (Höftberger et al., 2015).

In our case series, one patient had a history of infection in which HSV was detected in the CSF using NGS. The patient’s symptoms gradually recovered after antiviral and immunotherapy. We believe that antiviral also plays an important role in the recovery of the disease. Patients with anti-AMPAR encephalitis should be actively tested for infectious factors, as early diagnosis and treatment could potentially improve the disease outcome.

Anti-AMPAR encephalitis has been reported across a wide age range, primarily in middle-aged and older individuals (Wang et al., 2025; Zhu et al., 2024). Approximately half of the cases of anti-AMPAR encephalitis occur in individuals aged 50–70, with a higher incidence in females compared to males (Wang et al., 2025; Zhu et al., 2024). Zhu et al. reported that the average age of individuals in the tumor combination group was 52, which is significantly higher than the average age of 40 in the non-tumor group (Zhu et al., 2024). In our series of cases, two individuals developed the disease during middle age, and one at a younger age. However, none of them had tumors.

Clinical manifestations of anti-AMPAR encephalitis are associated with the distribution of AMPAR within the CNS. Considering that anti-AMPAR antibodies are extensively distributed throughout the CNS, patients may present with diffuse encephalitis (Höftberger et al., 2015). However, GluA1/2 and GluA2/3 are expressed at exceptionally high levels in the hippocampus and other limbic structures (Sprengel, 2006). Thus, anti-AMPAR encephalitis predominantly manifests clinically as LE. The most common clinical manifestation of anti-AMPAR encephalitis is cognitive dysfunction, with the loss of recent memory as the initial symptom in most patients, followed by subsequent progression to dementia in a few cases (Jia et al., 2021). Seizures are also a common clinical presentation, including various seizure types. Along with the typical LE symptoms, patients with anti-AMPAR encephalitis may exhibit rare symptoms, such as mood disorders (restlessness or reticence), urinary incontinence, sleep disorders, dysphagia, and autonomic dysfunction. Relevant literature has also reported that patients with anti-AMPAR encephalitis may present with stroke-like symptoms, such as weakness in one limb, as an initial manifestation (Höftberger et al., 2015). However, of the three patients in this case series, one presented with memory issues, while the other two presented with LE symptoms. None of them exhibited seizures. 90.5% of individuals with combined tumors experienced mental symptoms, including cognitive impairment and abnormal behavior, compared to 38.9% in the non-combined patients (Zhu et al., 2024). Patients with tumors may exhibit more complex symptoms, such as the combination of myasthenia gravis and thymoma (Zhang T. Y. et al., 2021).

Most patients with anti-AMPAR2 encephalitis exhibit varied cranial MRI manifestations, commonly presenting with unilateral or bilateral abnormalities in the medial temporal lobe, cingulate gyrus, and insula, as well as high signal on T2 fluid-attenuated inversion recovery (FLAIR) sequences. Moreover, involvement of the basal ganglia, cerebral cortex, and cerebellum has been reported. During long-term follow-up, hippocampal or cortical atrophy has been noted on cranial MRI (Zhang T. Y. et al., 2021). Additionally, some patients may exhibit clinical manifestations without any obvious abnormalities on cranial MRI (Zhang Z. et al., 2021; Zhang T. Y. et al., 2021). This finding may be attributed to low antibody titers and a transient antibody attack on the nerve conduction bundles, which can lead to ionic imbalances and consequent symptoms. In this case series, the main sites of the lesions in the patients in cases 1 and 3 were the cingulate gyrus, bilateral hippocampus, and cerebral cortex, which are the common lesion locations in this disease. Subsequent brain MRI revealed atrophy in the hippocampal or cortical regions. The cranial MRI of the patient in case 2 did not show any significant abnormalities, a relatively rare observation that may be linked to the lower antibody titer and milder clinical symptoms.

The presence of positive serum or CSF anti-AMPAR antibodies are necessary for the diagnosis of anti-AMPAR encephalitis, with antibodies against AMPAR1 and/or AMPAR2 being the most prevalent. Wang et al. studied 37 cases of anti-AMPAR encephalitis, discovering that 73% of the patients had antibodies in both serum and CSF, 11% had antibodies only in CSF, and 16% had antibodies only in serum, involving a case that was not discussed in the original literature (Wang et al., 2025). The sensitivity of CSF detection is higher, especially when the antibody titer is low; CSF is more likely to detect antibodies in such cases. Simple positive low serum titer requires careful interpretation, while a positive CSF result is more supportive of the diagnosis of anti-AMPAR encephalitis (Zhang T. Y. et al., 2021). The presence of double antibodies significantly supports the diagnosis of AMPAR encephalitis (Zhang T. Y. et al., 2021). Positive CSF antibodies suggest intrathecal synthesis, which more directly reflects the autoimmune attack on neurons. Serum positivity may indicate an active systemic immune response or disruption of the BBB, suggesting more extensive inflammatory damage. This damage may aggravate cognitive impairment or epileptic symptoms. However, a comprehensive judgment should be made in conjunction with clinical manifestations and other relevant examinations (Wang et al., 2021; Zhang T. Y. et al., 2021). In our cohort, both Case 1 and Case 3 revealed the presence of anti-AMPAR2 antibody in CSF and serum. In Case 1, the serum anti-AMPAR2 antibody titer was higher than that in the CSF, and the QALB was within the normal range, suggesting no significant BBB disruption. This finding implies that the observed serum/CSF anti-AMPAR2 antibody titer discrepancy may involve alternative mechanisms, such as transient BBB opening during acute inflammation or intrathecal antibody synthesis.

The relationship between antibody levels and disease severity is still not clear. Antibody levels might indicate the strength of an autoimmune reaction, yet they do not solely determine the severity of the disease. For instance, in paraneoplastic syndrome, elevated levels may suggest more complex immune issues (Ricken et al., 2021). Regarding patients with anti-AMPAR encephalitis, initial antibody levels do not significantly correlate with the severity of clinical symptoms (Wang et al., 2021). After immunotherapy, antibody titer reduction is often linked to symptom improvement, with CSF titer changes being more sensitive than those in serum (Wang et al., 2021). The reduction of CSF antibody levels following treatment is strongly linked to clinical improvements, such as better cognitive function and improved epilepsy management, and it may serve as a potential indicator of treatment effectiveness. Still, it needs to be evaluated in conjunction with MRI and neurological function scores (Zhang T. Y. et al., 2021). A lack of significant decrease in antibody titer or its continued positivity may indicate a poor treatment response or an increased likelihood of recurrence (Wang et al., 2021). Early immunotherapy can accelerate the drop in antibody titers and significantly improve the prognosis, while delayed intervention may result in slower antibody clearance and prolonged symptoms. Even when symptoms improve, immunotherapy should be continued if antibodies are not completely negative to prevent recurrence (Wang et al., 2021). Regular monitoring of antibody levels is advised to evaluate disease activity dynamically. Consistently high antibody levels or recurring symptoms are key indicators for starting second-line therapy (Wang et al., 2021). It is important to note that antibody titer predictions for prognosis are not definitive and should be evaluated with clinical factors such as tumor type and other health conditions (Huang et al., 2023). In our study group, the CSF titers of Case 1 and Case 3 were both 1:100, which was higher than that of Case 2, and the symptoms of both cases were more severe. However, the recovery of Case 3 was worse. Case 2 had the lowest CSF antibody titer, the mildest symptoms, and the best recovery. Further testing for CSF antibody will be recommended for these patients as needed.

Anti-alpha-amino-3-hydroxy-5-methyl-4-isoxazolepropionic acid receptor 1 and AMPAR2 mediate synaptic plasticity and stability, respectively. The presence of positive double antibodies may cause a reduction in receptor density, impair synaptic function, and lead to abnormal neuronal excitability, which may result in more severe cognitive decline and epilepsy. In our study, Case 2 revealed the presence of the anti-AMPAR1 and the anti-AMPAR2 antibodies in the CSF. The patient experienced only memory decline and made a full recovery following treatment, which may be related to the relatively low antibody titer. In addition, the influence of double antibodies on the GluA1/GluA2 ratio may also affect the disease’s severity. The clinical similarities and differences between patients with both antibodies and those with only one antibody are still not well understood.

Treatment for anti-AMPAR encephalitis can follow the standard procedures for AE. It is recommended to start first-line treatment immediately after ruling out infectious causes. Patients with severe AE should start treatment with high doses of corticosteroids along with IVIG or plasma exchange (PLEX), while steroid monotherapy may be an option for mild to moderate cases. Second-line therapeutic interventions should be administered to all patients with severe AE who do not demonstrate clinical improvement or experience exacerbation of symptoms within 5–10 days following the initiation of first-line treatment regimens. For patients with mild to moderate disease severity, this period may extend to 2–4 weeks. Because anti-AMPAR antibodies target cell surface antigens, rituximab is the preferred second-line treatment for anti-AMPAR encephalitis. Cyclophosphamide treatment can also be considered as a second-line option for patients who do not respond to first-line therapies. If the second-line treatment is unsuccessful, corticosteroids, IVIG, and PLEX can be used, as long as they were not part of the initial therapy. For patients who do not respond to second-line treatments, third-line and experimental immunotherapies such as tocilizumab and bortezomib might be options (Hahn et al., 2024). In our case series, the initial treatment of the patient in case 3 with first-line immunotherapy yielded a poor therapeutic effect. The disease recurred, prompting the addition of rituximab at a subsequent stage, after which the disease did not reappear. In contrast, the other two patients were administered only first-line immunotherapy. If the initial treatment doesn’t work, it’s advised to start the second-line treatment promptly.

For patients with concurrent tumors, early intervention in the tumors is crucial for improving the neurological outcomes of patients with anti-AMPAR encephalitis. It is recommended to advance immunotherapy and tumor treatment together. Research indicates that the favorable prognosis rate in the combined tumor group (41.7%) is much lower compared to the non-tumor group (83.3%), which may be connected to the continuous production of pathogenic antibodies or the initiation of immune evasion mechanisms in the tumor microenvironment (Zhu et al., 2024). The median follow-up duration for the combined tumor group was only 9.8 months, suggesting that disease progression in these patients was more aggressive. Clinical data indicated that complications related to tumors, rather than neurological damage, were the primary cause of death (Zhu et al., 2024). The death rate for lung cancer patients reaches 47.1%, largely due to delayed diagnosis or respiratory complications. Thymic tumors have a 20% mortality rate, and their poor prognosis may be associated with coexisting myasthenia gravis or strong local invasiveness (Huang et al., 2023). It is worth noting that tumor recurrence may trigger the reactivation of encephalitis (Zhang T. Y. et al., 2021). Patients with anti-AMPAR encephalitis are recommended to undergo tumor screenings. For patients diagnosed with tumors, it is essential to establish a multidisciplinary diagnosis and treatment team to coordinate the timing of neuroimmunotherapy and tumor-specific treatments to improve long-term survival (Zhang T. Y. et al., 2021). However, no comorbid tumors were detected in our three patients. This could be due to a shorter follow-up duration or a smaller number of patients. Nevertheless, clinicians should continue to screen for tumors in patients with anti-AMPAR2 encephalitis, even during later follow-ups (Lin et al., 2023).

It is suggested to provide intravenous acyclovir treatment and to begin first-line immunotherapy concurrently for AE following HSV infection. In addition, swiftly beginning second-line immunosuppressive treatment for patients who do not respond well to initial treatment (Sun et al., 2023).

The limitation of our paper is that the number of anti-AMPAR2 encephalitis patients is small, which introduces bias. Additionally, the clinical manifestations, treatment, and prognosis can only reflect the characteristics of this specific population. Nevertheless, this data can enhance our understanding of anti-AMPAR2 encephalitis, particularly the cases caused by viral infections, and active antiviral treatment may help improve prognosis.

## 5 Conclusion

Anti-AMPAR2 encephalitis is a rare AE with heterogeneous clinical features. The disease frequently manifests with symptoms affecting memory and the limbic system. Given the possibility of viral infections triggering the disease, it is important to quickly perform etiological testing on CSF, as strong antiviral treatment may significantly improve recovery outcomes. Furthermore, tracking CSF antibody titers over time during treatment could enhance the effectiveness of disease assessment. For patients who don’t respond effectively to initial treatment, timely initiation of second-line therapy is essential. Lastly, given that anti-AMPAR encephalitis can occur with combined malignancies, regular tumor screening, even during follow-ups, is warranted.

## Data Availability

The original contributions presented in this study are included in this article/supplementary material, further inquiries can be directed to the corresponding author/s.

## References

[B1] Ban Y. Yang X. Tan D. Gong C. Gao Y. Yuan J. (2024). Sorbs2 regulates seizure activity by influencing AMPAR-mediated excitatory synaptic transmission in temporal lobe epilepsy. *Neurochem. Int.* 176:105727. 10.1016/j.neuint.2024.105727 38555055

[B2] Boulter J. Hollmann M. O’Shea-Greenfield A. Hartley M. Deneris E. Maron C. (1990). Molecular cloning and functional expression of glutamate receptor subunit genes. *Science* 249 1033–1037. 10.1126/science.2168579 2168579

[B3] Dalmau J. Tüzün E. Wu H. Y. Masjuan J. Rossi J. E. Voloschin A. (2007). Paraneoplastic anti-N-methyl-D-aspartate receptor encephalitis associated with ovarian teratoma. *Ann. Neurol.* 61 25–36. 10.1002/ana.21050 17262855 PMC2430743

[B4] Davies G. Irani S. R. Coltart C. Ingle G. Amin Y. Taylor C. (2010). Anti-N-methyl-D-aspartate receptor antibodies: A potentially treatable cause of encephalitis in the intensive care unit. *Crit. Care Med.* 38 679–682. 10.1097/CCM.0b013e3181cb0968 20016378

[B5] Gnann J. W. Whitley R. J. (2017). Herpes simplex encephalitis: An update. *Curr. Infect. Dis. Rep.* 19:13. 10.1007/s11908-017-0568-7 28251511

[B6] Graus F. Delattre J. Y. Antoine J. C. Dalmau J. Giometto B. Grisold W. (2004). Recommended diagnostic criteria for paraneoplastic neurological syndromes. *J. Neurol. Neurosurg. Psychiatry* 75 1135–1140. 10.1136/jnnp.2003.034447 15258215 PMC1739186

[B7] Graus F. Titulaer M. J. Balu R. Benseler S. Bien C. G. Cellucci T. (2016). A clinical approach to diagnosis of autoimmune encephalitis. *Lancet Neurol.* 15 391–404. 10.1016/S1474-4422(15)00401-9 26906964 PMC5066574

[B8] Guan H. Z. Ren H. T. Cui L. Y. (2016). Autoimmune encephalitis: An expanding frontier of neuroimmunology. *Chin. Med. J. (Engl)* 129 1122–1127. 10.4103/0366-6999.180514 27098800 PMC4852682

[B9] Guasp M. Landa J. Martinez-Hernandez E. Sabater L. Iizuka T. Simabukuro M. (2021). Thymoma and autoimmune encephalitis: Clinical manifestations and antibodies. *Neurol. Neuroimmunol. Neuroinflamm.* 8:e1053. 10.1212/NXI.0000000000001053 34301822 PMC8312280

[B10] Hahn C. Budhram A. Alikhani K. AlOhaly N. Beecher G. Blevins G. (2024). Canadian consensus guidelines for the diagnosis and treatment of autoimmune encephalitis in adults. *Can. J. Neurol. Sci.* 10.1017/cjn.2024.16 [Epub ahead of print]. 38312020

[B11] Höftberger R. van Sonderen A. Leypoldt F. Houghton D. Geschwind M. Gelfand J. (2015). Encephalitis and AMPA receptor antibodies: Novel findings in a case series of 22 patients. *Neurology* 84 2403–2412. 10.1212/WNL.0000000000001682 25979696 PMC4478035

[B12] Huang Y. Zhou M. Zhou J. Wu B. Yang X. Min W. (2023). Anti-alpha-amino-3-hydroxy-5-methyl-4-isoxazolepropionic acid receptor encephalitis developed after ovarian cancer cytoreduction surgery: A case report and literature review. *BMC Womens Health* 23:507. 10.1186/s12905-023-02636-1 37735388 PMC10512534

[B13] Jahanban-Esfahlan R. Seidi K. Majidinia M. Karimian A. Yousefi B. Nabavi S. M. (2019). Toll-like receptors as novel therapeutic targets for herpes simplex virus infection. *Rev. Med. Virol.* 29:e2048. 10.1002/rmv.2048 31265195

[B14] Jia Y. Li M. Wang H. Zhang M. Wang Y. (2021). The peculiar clinical symptoms and treatment of limbic encephalitis associated with AMPA receptor antibody. *Eur. Neurol.* 84 206–211. 10.1159/000515592 33857949

[B15] Keinänen K. Wisden W. Sommer B. Werner P. Herb A. Verdoorn T. A. (1990). A family of AMPA-selective glutamate receptors. *Science* 249 556–560. 10.1126/science.2166337 2166337

[B16] Lai M. Hughes E. G. Peng X. Zhou L. Gleichman A. J. Shu H. (2009). AMPA receptor antibodies in limbic encephalitis alter synaptic receptor location. *Ann. Neurol.* 65 424–434. 10.1002/ana.21589 19338055 PMC2677127

[B17] Liao Y. Wen L. Zheng R. Shen Y. Ha T. A. Lin M. (2025). Novel perspectives focused on the relationship between herpesvirus encephalitis and Anti-GFAP-Antibody-Positive astrocytopathy. *Mol. Neurobiol.* 62 6179–6194. 10.1007/s12035-024-04660-0 39731639

[B18] Lin J. Wang J. Li J. (2023). Patient characteristics and outcome in patients with anti-alpha-amino-3-hydroxy-5-methyl-4-isoxazolepropionic acid receptor (AMPAR) encephalitis. *Neurol. Sci.* 44 3253–3259. 10.1007/s10072-023-06769-x 37010671

[B19] Linnoila J. J. Binnicker M. J. Majed M. Klein C. J. McKeon A. C. S. F. (2016). herpes virus and autoantibody profiles in the evaluation of encephalitis. *Neurol. Neuroimmunol. Neuroinflamm.* 3:e245. 10.1212/NXI.0000000000000245 27308306 PMC4897981

[B20] Mayer M. L. (2005). Glutamate receptor ion channels. *Curr. Opin. Neurobiol.* 15 282–288. 10.1016/j.conb.2005.05.004 15919192

[B21] Peng X. Hughes E. G. Moscato E. H. Parsons T. D. Dalmau J. Balice-Gordon R. J. (2015). Cellular plasticity induced by anti-α-amino-3-hydroxy-5-methyl-4-isoxazolepropionic acid (AMPA) receptor encephalitis antibodies. *Ann. Neurol.* 77 381–398. 10.1002/ana.24293 25369168 PMC4365686

[B22] Ren H. Fan S. Zhao Y. Guan H. (2021). The changing spectrum of antibody-mediated encephalitis in China. *J. Neuroimmunol.* 361:577753. 10.1016/j.jneuroim.2021.577753 34739913

[B23] Ricken G. Zrzavy T. Macher S. Altmann P. Troger J. Falk K. K. (2021). Autoimmune global amnesia as manifestation of AMPAR encephalitis and neuropathologic findings. *Neurol. Neuroimmunol. Neuroinflamm.* 8:e1019. 10.1212/NXI.0000000000001019 34016735 PMC8142837

[B24] Samad N. Wong J. (2018). Anti-AMPA receptor encephalitis associated with medullary thyroid cancer. *BMJ Case Rep.* 2018:bcr2018225745. 10.1136/bcr-2018-225745 30150348 PMC6119387

[B25] Segal Y. Rotschild O. Mina Y. Maayan Eshed G. Levinson T. Paran Y. (2024). Epidemiology of autoimmune encephalitis and comparison to infectious causes-Experience from a tertiary center. *Ann. Clin. Transl. Neurol.* 11 2337–2349. 10.1002/acn3.52147 39030965 PMC11537142

[B26] Sprengel R. (2006). Role of AMPA receptors in synaptic plasticity. *Cell Tissue Res.* 326 447–455. 10.1007/s00441-006-0275-4 16896950

[B27] Suh-Lailam B. B. Haven T. R. Copple S. S. Knapp D. Jaskowski T. D. Tebo A. E. (2013). Anti-NMDA-receptor antibody encephalitis: Performance evaluation and laboratory experience with the anti-NMDA-receptor IgG assay. *Clin. Chim. Acta* 421 1–6. 10.1016/j.cca.2013.02.010 23454475

[B28] Sun S. Ren J. Zhong Z. Ma X. Shang D. Su C. (2023). Case report: Overlapping anti-AMPAR encephalitis with anti-IgLON5 disease post herpes simplex virus encephalitis. *Front. Immunol.* 14:1329540. 10.3389/fimmu.2023.1329540 38259458 PMC10800422

[B29] Wang K. Shi Y. Du Q. Zhang R. R. Wu H. Qiao S. (2021). Clinical review and prognostic analysis of α-Amino-3-Hydroxy-5-Methyl-4-Isoxazole propionate receptor-associated encephalitis. *Front. Neurol.* 12:665229. 10.3389/fneur.2021.665229 34054708 PMC8155358

[B30] Wang X. Zhao C. Chen Q. Yu W. Zhao S. Wang P. (2025). Anti-α-amino-3-hydroxy-5-methyl-4-isoxazolepropionic acid receptor 2 encephalitis with olfactory hallucination: A case report and literature review. *Front. Immunol.* 16:1444053. 10.3389/fimmu.2025.1444053 40051626 PMC11882523

[B31] Zhang T. Y. Cai M. T. Zheng Y. Lai Q. L. Shen C. H. Qiao S. (2021). Anti-Alpha-Amino-3-Hydroxy-5-Methyl-4-Isoxazolepropionic acid receptor encephalitis: A review. *Front. Immunol.* 12:652820. 10.3389/fimmu.2021.652820 34093540 PMC8175895

[B32] Zhang Z. Fan S. Ren H. Zhou L. Guan H. (2021). Clinical characteristics and prognosis of anti-alpha-Amino-3-Hydroxy-5-Methyl-4-Isoxazolepropionic acid receptor encephalitis. *BMC Neurol.* 21:490. 10.1186/s12883-021-02520-1 34915865 PMC8678635

[B33] Zhu Q. Y. Liang D. X. Feng F. Teng J. F. (2024). [Characteristic analysis of autoimmune encephalitis with antibodies against the α-amino-3-hydroxy-5-methyl-4-isoxazolepropionic acid receptor]. *Zhonghua Yi Xue Za Zhi* 104 3681–3684. 10.3760/cma.j.cn112137-20240409-00830 39428218

